# Pulmonary mucormycosis in an immunocompetent young female: a case report and literature review

**DOI:** 10.3389/fmed.2024.1491489

**Published:** 2024-12-24

**Authors:** Xun Zhang, Zhenbin Wu, Shifeng Shao

**Affiliations:** Department of ICU, Daping Hospital, Army Medical University, Chongqing, China

**Keywords:** pulmonary mucormycosis, fungal infection, airway collapse, mediastinal infection, pneumonia

## Abstract

Mucormycosis is considered a rare but highly lethal fungal infection, often occurring in patients with poorly controlled diabetes or immunosuppression. Pulmonary mucormycosis progresses rapidly and is often associated with pulmonary infarction and hemoptysis. In this case report, we presented a young, immunocompetent female patient with newly diagnosed diabetes who was diagnosed early with *Rhizopus delemar* infection through metagenomic next-generation sequencing. Despite early diagnosis, the infection progressed rapidly, invading the tracheal cartilage and upper mediastinal soft tissue, ultimately leading to the patient’s unfortunate demise.

## Introduction

Mucormycosis is a life-threatening opportunistic infection caused by fungi of the order Mucorales. It primarily affects individuals with poorly controlled diabetes or immunosuppression (1–4). Fungal spores can gain access to the respiratory tract via inhalation, reach the skin by means of direct inoculation at the site of a wound, or enter the body through ingestion via the gastrointestinal tract. Once inside the host, the spores germinate into hyphae, which invade blood vessels, leading to tissue infarction and necrosis, and can disseminate hematogenously to involve multiple organs (4, 5). Rhino-orbito-cerebral mucormycosis (ROCM) and pulmonary mucormycosis (PM) are the most common clinical manifestations, with the mortality rate of PM ranging from 40 to 80% (3, 6–8). Reports of PM in patients with newly diagnosed diabetes are rare, and cases that rapidly progress to involve both the trachea and mediastinum are even rarer (3). In this case report, we present a 19-year-old previously healthy female patient with newly diagnosed diabetes who developed PM caused by *Rhizopus delemar*. We achieved an early diagnosis of PM through mNGS. Within two weeks, the extensive pulmonary infarction, severe airway collapse, upper mediastinal infection, and eventually, massive hemoptysis due to multiple pulmonary vascular hemorrhages, leading to death.

## Case report

**Figure 1:**
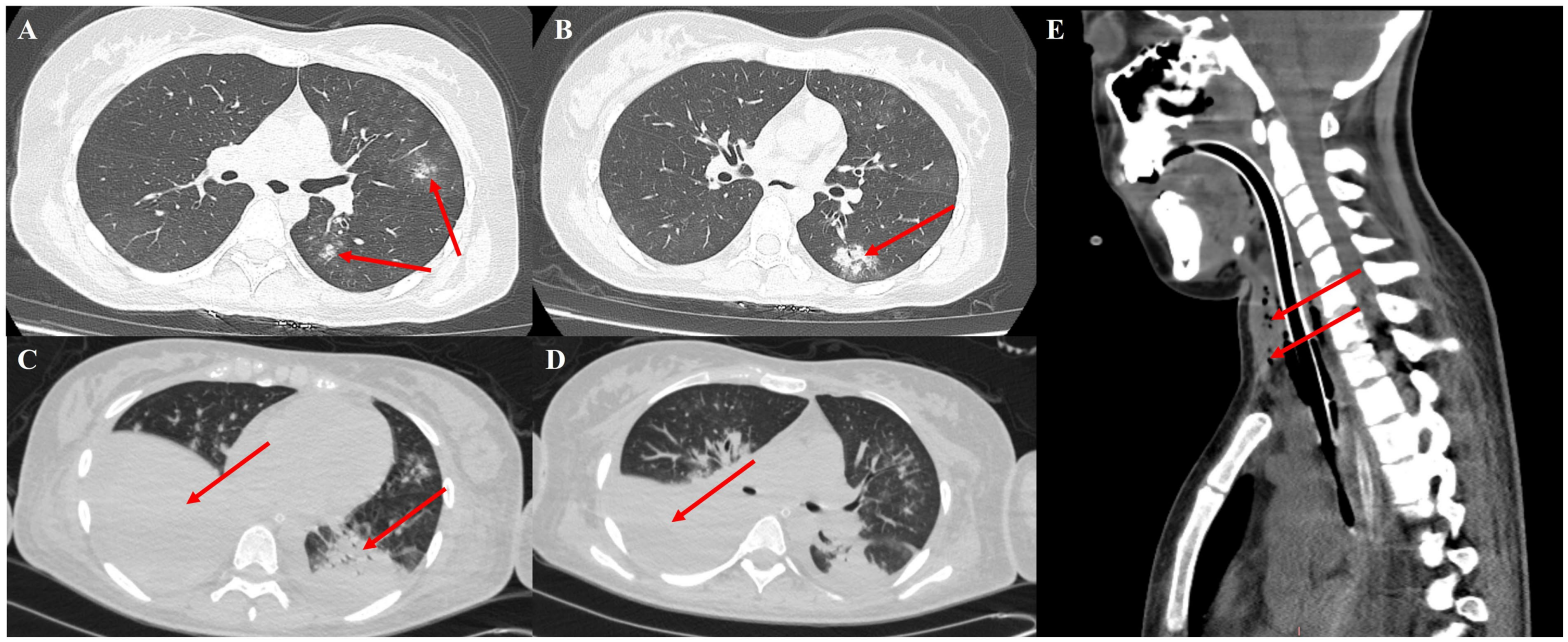
**(A,B)** On the first day of admission, a CT scan revealed multiple scattered patchy opacities in the left lung. **(C,D)** By day 5, the scan showed extensive consolidation and atelectasis in the bilateral lower lobes, with a large amount of pleural effusion on both sides. **(E)** By day 5, sagittal views indicated soft tissue infection in the upper mediastinum secondary to fungal infection, along with an air-crescent sign.

**Figure 2:**
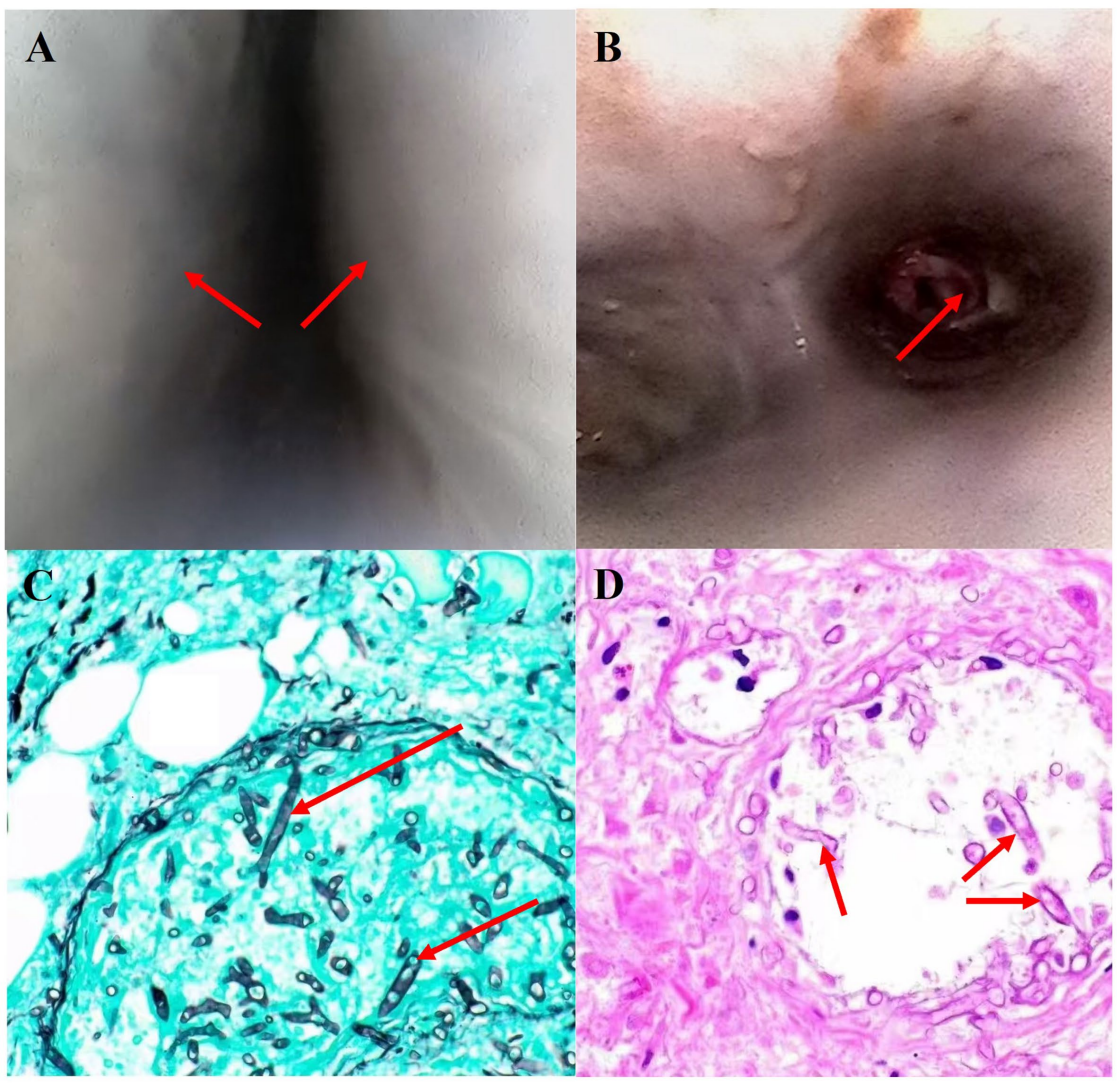
**(A,B)** Bronchoscopy revealed severe airway narrowing and mucosal necrosis. Histopathological examination of necrotic mediastinal soft tissue, stained with Grocott’s methenamine silver [**(C)** ×200] and hematoxylin and eosin [**(D)** ×200] showed hyphae of broad, non-septate hyphae, irregularly branching.

The patient was a 19-year-old female with no previous comorbidities who presented with a four-day history of cough and chest tightness. She had a previous history of polydipsia, polyuria, and polyphagia but had not been diagnosed with diabetes and had not taken glucocorticoids. She also showed normal pulmonary function in the past. She initially sought treatment at a community hospital, where she was diagnosed with community-acquired pneumonia and treated with levofloxacin. Despite this, her condition progressively worsened. One day before admission, her symptoms escalated, and she developed dyspnea and altered consciousness. Upon admission, physical examination revealed that the patient was in a state of shallow coma, with a temperature of 38.5°C, pulse rate of 116 beats per minute, and blood pressure of 146/89 mmHg. With 5 L/min of oxygen delivered by face mask, her pulse oximetry reading was 85%. Auscultation revealed markedly diminished breath sounds in both lower lungs. Laboratory tests showed a white blood cell count of 25.82 × 10^9^/L, with 82.9% neutrophils, a procalcitonin (PCT) level of 15.6 ng/mL, C-reactive protein (CRP) of 25 mg/L, glycated hemoglobin (HbA1c) of 15%, and urine ketones +++. The test results of galactomannan and 1,3-*β* -D - glucan are both negative. Immunological tests showed no significant abnormalities. A chest CT scan performed upon admission revealed multiple scattered patchy opacities in the left lung (Figures 1A,B). With informed consent from the patient’s family, tracheal intubation and mechanical ventilation were initiated. Flexible bronchoscopy showed extensive mucosal edema, severe airway narrowing, and grayish-white mucosal walls (Figures 2A,B; Supplementary Videos S1, S2). Bronchoalveolar lavage fluid was collected for culture and metagenomic next-generation sequencing (mNGS). Given the patient’s severe community-acquired pneumonia, we empirically adopted piperacillin-sulbactam for anti-infection treatment. On the second day after admission, the BALF smear and found fungal spores and hyphae under the microscope and mNGS revealed a high number of *Rhizopus delemar* sequences and a small amount of *Aspergillus* species, consistent with subsequent microbiological culture results. The antifungal therapy was immediately adjusted to intravenous isavuconazole (On admission, the patient showed a creatinine level of 252 umol/L and urea nitrogen at 12 mmol/L, with a mere 500 mL of 24-h urine volume post-admission. Thus, continuous renal replacement therapy was initiated promptly. In light of the patient’s renal function condition, we refrained from using amphotericin B intravenously) and nebulization of amphotericin B. The trachea exhibited a fusiform appearance (Supplementary Videos S3, S4), and the endotracheal tube balloon could no longer seal properly against the tracheal wall, resulting in minor air leakage during mechanical ventilation. On the fifth day of hospitalization, a repeat chest imaging revealed extensive consolidation and atelectasis of the bilateral lower lobes, predominantly on the right side. Sagittal views showed tissue necrosis and the presence of an air-crescent sign in the anterior mediastinum, indicative of fungal proliferation (Figures 1C–E). Upon the seventh day of admission, A fiberoptic bronchoscopy examination was conducted. It was revealed that the airway had become more constricted as compared to that on the fifth day (Supplementary Video S5). The results of the bronchoalveolar lavage fluid culture indicated the growth of *Rhizopus*. In collaboration with an otolaryngologist, a bedside tracheostomy was performed using an extended tracheostomy tube. Tissue from the cervical subcutaneous area was biopsied (Figures 2C,D), revealing necrotic tissue and abundant Rhizopus hyphae and spores. Despite aggressive antifungal therapy, the patient’s condition rapidly deteriorated. Tragically, on the twelfth day of hospitalization, the patient suffered massive hemoptysis, which resulted in life-threatening airway bleeding. The severity of the hemorrhage precluded the timely placement of a double-lumen endotracheal tube, and the patient ultimately succumbed to exsanguination.

## Discussion

Mucormycosis is a highly angio-invasive fungal infection caused by fungi of the order Mucorales. Although rare, mucormycosis has been recognized for its exceedingly high mortality rate, which has regrettably not shown significant improvement in recent years (9, 10). We reviewed 14 PM cases in immunocompetent hosts published in PubMed from 2000 to 2024. The review summarizes the age, gender, presentations, treatment, diagnosis method, and final outcome (Table 1). The presenting age ranged between 19 and 67 years. Among the cases where the pathogen can be clearly identified, the genus *Rhizopus* is the most common pathogen, and among them, *Rhizopus microsporus* is the most common species (11–14). In these previously published case reports, except four reported cases in which certain patients exhibited a specific inhalation history, the mechanism underlying mucor infection in the majority of immunocompetent individuals indeed remains elusive and poorly understood (12, 15, 16). Although these individuals did not have concurrent severe immunodeficiency, a significant proportion of six patients nonetheless died.

**Table 1:** Summary of previous literature on pulmonary mucormycosis in immunocompetent patients published in PubMed from 2000 to 2024.

Author	Age/gender	Pathogen	Presentation	Treatment	Diagnosis method	Outcome
He et al. (15)	32/male	Absidia	Cough, fever, dyspnea, hemoptysis	Posaconazole, amphotericin B	BALF culture	Live
Balta C et al. (19)	37/female	NA	Hemoptysis	Surgical resection	pathological	Live
Lee et al. (27)	52/female	NA	Hemoptysis	Amphotericin B, posaconazole	CT-guided percutaneous biopsy	Live
Yang et al. (28)	46/female	NA	Pancoast syndrome, bone destruction of ribs	Posaconazole	CT-guided percutaneous biopsy	Died
Rouientan et al. (29)	65/male	NA	Cough, chest pain	Liposomal amphotericin B	Transbronch-ial biopsy	Died
Huang et al. (11)	60/F	Rhizomucor pusillus	Bipolar disorder, hypothyroidism, acute liver failure	Liposomal amphotericin B	BALF culture	Died
Acharya et al. (30)	44/M	NA	Fever, cough, dyspnea, chest pain	Amphotericin B deoxycholate	Biopsy	NA
Guo et al. (12)	43/M	Rhizopus microsporus	Cough	Surgical resection	mNGS	Live
Manjunath et al. (31)	62/M	NA	Dry cough, fever, chills	Liposomal amphotericin B, posaconazole, micafungin, surgical resection	Transbronch-ial biopsy	Died
Al-Tikrity et al. (13)	20/M	Rhizopus	Ough, sore throat, fever	Amphotericin B, caspofungin	BALF culture	Died
Harada et al. (32)	67/M	Cunninghamella bertholletiae	progressive dyspnea, productive cough, moderate fever	Liposomal amphotericin B	PCR based direct sequencing	Died
Song et al. (16)	56/M	Lichtheimia corymbifera	lost consciousness due to the inhalation of biogas	Amphotericin B	BALF culture	Live
	34/M	Lichtheimia corymbifera		Amphotericin B	BALF culture	Live
Chen et al. (14)	19/F	Rhizopus microsporus	Hemoptysis, irritative cough	Amphotericin B, posaconazole	mNGS	Live

M, male; F, female; NA, not available; mNGS, metagenomic next-generation sequencing; BALF, Bronchoalveolar lavage Fluid. PCR, polymerase chain reaction.

The most common forms are ROCM and PM, which are classified based on clinical presentation and anatomical involvement. ROCM is most frequently observed in patients with poorly controlled diabetes, while PM is more common in immunocompromised individuals (4, 6, 17). However, this case is noteworthy as the patient was previously healthy, and PM was diagnosed concomitantly with the first diagnosis of diabetes. Some cases of PM have also been reported in patients without clear immunodeficiency or diabetes (18, 19). Previous studies have shown that the average age of mucormycosis patients is 51 years, with the youngest being 39 years (6). In two additional retrospective studies dedicated to pulmonary mucormycosis, the mean ages of onset were determined to be 58 and 55 years respectively, with the minimum age recorded as 43 years (17, 20). However, this case report described a patient who was only 19 years old, challenging our understanding of the age range affected by mucormycosis. The complexity of the affected population and the individual variability in presentation necessitate a high index of suspicion for mucormycosis in different patient groups.

Diagnosing mucormycosis has traditionally been challenging. Common serological tests for fungal infections, such as *β*-D-glucan and galactomannan antigen, are typically negative in mucormycosis because the causative fungi lack or have minimal amounts of these cell wall components. Additionally, among patients with confirmed mucormycosis, only 15–25% of cases have positive fungal cultures (21), and the slow growth and high risk of contamination associated with fungal cultures further complicate diagnosis. During the waiting period for culture results, the patient’s condition may rapidly deteriorate, leading to delayed treatment. Previous reports have highlighted the critical role of mNGS in diagnosing ROCM (22). In this case, mNGS was also instrumental in confirming the diagnosis of PM early in the disease course, allowing for timely treatment initiation. This report underscores the importance of mNGS testing in patients with a high suspicion of PM.

Typical PM findings include bronchial mucosal swelling, congestion, and diffuse coverage with white, cheese-like material, consistent with the bronchoscopy findings in this case (23, 24). Farid et al. also reported the occurrence of tracheoesophageal fistulas in PM patients (25). In our case, severe necrosis of the tracheal cartilage rings led to complete tracheal collapse into a fusiform shape. Moreover, the anterior mediastinum showed significant soft tissue destruction due to fungal growth, evidenced by the air-crescent sign. Such extensive tissue damage from fungal infection may be attributed to the highly invasive nature of Mucorales, suggesting that we may often underestimate the invasive potential of PM. The guideline recommends that anti-mucormycosis treatment should be initiated as early as possible when the patient is immunocompromised and there is a high suspicion of mucormycosis infection. However, although this patient has newly diagnosed diabetes, based on a comprehensive review of multiple clinical test results of immune function, no manifestations of immunocompromise were detected (26). Therefore, an attempt to attain a diagnosis should be made at the time of initiation of therapy to avoid delaying treatment. For patients in whom tracheal collapse due to infection is detected via fiberoptic bronchoscopy, especially those with concurrent diabetes mellitus, a high level of alertness should be maintained regarding the potential occurrence of pulmonary mucormycosis infection.

Despite being a rare invasive fungal disease, PM presents significant challenges due to its high mortality rate and rapid progression. The age at onset may be younger than previously expected. mNGS is recommended as a rapid diagnostic tool in cases of high suspicion of PM. Clinicians should be aware of the potential for rapid airway cartilage damage, tracheal collapse, and mediastinal infection in these patients. Recognizing the complex manifestations of PM and diagnosing the condition promptly may gradually improve the success rate of treating PM.

## Data Availability

The original contributions presented in the study are included in the article/Supplementary material, further inquiries can be directed to the corresponding author.
