# Supplementary material for: Pulmonary mucormycosis in an immunocompetent young female: a case report and literature review
Source: Front Med (Lausanne). 2024 Dec 24;11:1491489. doi: 10.3389/fmed.2024.1491489 (PMC11703827; doi:10.3389/fmed.2024.1491489)
Supplement: Supplementary file 1 [file suppl_fig_1.pdf]

Supplementary Figure 1. Admission (day)

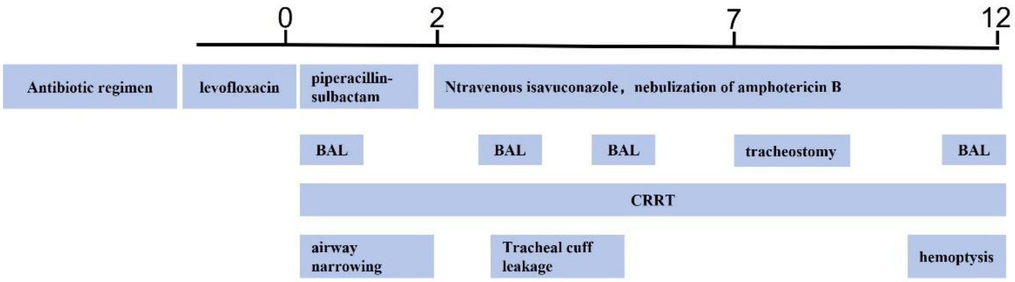

BAL, Bronchoalveolar Lavage via Fiberoptic Bronchoscopy; CRRT, Continuous Renal Replacement Therapy.
